# Stacking stability of C_2_N bilayer nanosheet

**DOI:** 10.1038/s41598-019-43363-8

**Published:** 2019-05-02

**Authors:** Klichchupong Dabsamut, Jiraroj T-Thienprasert, Sirichok Jungthawan, Adisak Boonchun

**Affiliations:** 1 Department of Physics, Faculty of Science, Kasetsart University, Bangkok, 10900 Thailand; 2 Thailand Center of Excellence in Physics, Commission on the Higher Education, 328 Si Ayutthaya Road, Bangkok, 10400 Thailand; 3 School of Physics, Institute of Science, and Center of Excellence in Advanced Functional Materials, Suranaree University of Technology, Nakhon Ratchasima, 30000 Thailand

**Keywords:** Electronic properties and materials, Two-dimensional materials

## Abstract

In recent years, a 2D graphene-like sheet: monolayer C_2_N was synthesized via a simple wet-chemical reaction. Here, we studied the stability and electronic properties of bilayer C_2_N. According to a previous study, a bilayer may exist in one of three highly symmetric stacking configurations, namely as AA, AB and AB′-stacking. For the AA-stacking, the top layer is directly stacked on the bottom layer. Furthermore, AB- and AB′-stacking can be obtained by shifting the top layer of AA-stacking by **a**/3-**b**/3 along zigzag direction and by **a**/2 along armchair direction, respectively, where a and b are translation vectors of the unit cell. By using first-principles calculations, we calculated the stability of AA, AB and AB′-stacking C_2_N and their electronic band structure. We found that the AB-stacking is the most favorable structure and has the highest band gap, which appeared to agree with previous study. Nevertheless, we furthermore examine the energy landscape and translation sliding barriers between stacking layers. From energy profiles, we interestingly found that the most stable positions are shifted from the high symmetry AB-stacking. In electronic band structure details, band characteristic can be modified according to the shift. The interlayer shear mode close to local minimum point was determined to be roughly 2.02 × 10^12^ rad/s.

## Introduction

Since graphene was discovered^1–5^, two-dimensional (2D) materials have attracted tremendous attention due to their novel electric, thermal, mechanical and optoelectronic properties^6–8^. The intrinsic zero-band gap of graphene is, however, unsuitable for many nanoelectronic device applications^1^. A number of modifications have been demonstrated to open a gap in graphene, such as doping and truncating to nanoribbons^9–14^. Other 2D materials have also been identified which possess a band gap, such as hexagonal boron nitride (h-BN) monolayer^15,16^, graphitic carbon nitride (C_3_N_4_)^17^, transition-metal dichalcogenides (TMDs)^18^, group-IV (silicene and germanene)^19^, group-V (phosphorene)^20^, group III–V (such as monolayer GaAs, InN and AlN)^21,22^ and especially molybdenum disulfide (MoS_2_)^23^. Recently, a monolayer carbon with nitrogen named C_2_N was synthesized via a bottom-up wet-chemical reaction^24^. C_2_N possesses an optical band gap of 1.96 eV which can be used for photocatalytic and electronic devices. Consequently, it is worthwhile to study their physical and electronic properties in order to gain a deeper understanding of this promising material for nanoelectronic devices.

The physical and electronic properties of C_2_N have received considerable attention in terms of density functional theory (DFT) calculations since it was synthesized^24^. Zhang *et al*.^25^ performed DFT-GGA calculations to investigate the physical and electronic structures of monolayer and bilayer C_2_N. In monolayer, they found that the optimized lattice parameter of C_2_N is a = b = 8.330 Å, two different types of C-C bonds are 1.429 and 1.470 Å, and the C-N bond is 1.336 Å. In electronic structure, the calculated band gap is 1.66 eV at the Γ point in DFT-GGA calculation^24–26^. As commonly known, DFT-GGA severely underestimate band gap attributed to the self-interaction error (SIE). Zhang *et al*. also^25^ performed band structure calculation using the hybrid functional (HSE). This calculated HSE band gap is 2.47 eV^25^ which is much higher than 1.96 eV of the experimental value^24^. In their research^25^, they are the first group who reported the structure and the stability of bilayer C_2_N, which provides three high-symmetry stacking orders (AA, AB and AB′-stacking) with different energy gaps. However, to identify the most favorable configuration, high-symmetry configuration is insufficient. Asymmetric configurations must also be taken into account. In fact, this issue is very important for evaluating the stacking stability of the bilayers. For example in MoS_2_, Tao Peng *et al*.^27^ provided the conclusion that the high symmetry stacking order with Mo and S superposed from both layers is the most stable stacking order. Similarly, bilayer h-BN gives the minimum energy sliding path only in AA′ high symmetry stacking with an energy barrier of 3.4 meV per atom^28^. Obviously, in most typical bilayer systems, the most stable configurations involve high symmetry or nearly high symmetry stacking order. However, in bilayer silicene, Huixia Fu *et al*.^29^ found several meta-stable configurations which are slightly shifted from the high symmetry AA-stacking structure, namely slide-2AA.

Here, we performed first-principles calculations to investigate the stability and electronic structure of bilayer C_2_N based on density functional theory (DFT). For a benchmark, we first studied the electronic properties of the monolayer with DFT-GGA and found that monolayer C_2_N has a direct band gap of 1.665 eV at Γ points, which agrees with previous works^24,25^. The relaxed structure and covalent bonds are agreed with previous work^25^. For bilayer calculations, the stability of symmetric and non-symmetric configuration of bilayer C_2_N were explored. Interestingly, we found that the high symmetry AB-configuration of bilayer C_2_N reported in previous study^25^ is not the most favorable structure. The most stable stacking is slightly shifted from AB-configuration. In this work, the stability and energy barriers have been also identified.

## Computational Method

Our calculations were based on the DFT as implemented in the Vienna ab-initio simulation package (VASP)^30,31^. The projector-augmented wave (PAW) method were used^32^ to treat the core electrons. For monolayer calculation, in order to obtain a more accurate description of the electronic states, the optimized lattice parameter and band structures were calculated using the hybrid functional (HSE). For bilayer calculation, The generalized gradient approximation of Perdew, Burke, and Ernzerhof (GGA-PBE) were used. The kinetic energy cut-off was set to 520 eV for the plane-wave expansions. The gamma centred 5 × 5 × 1 and 7 × 7 × 1 k-point sampling are adopted for monolayer and bilayer calculation parts, respectively. The total energy calculations were performed using the 18-atom C_2_N unit cell and 36-atom C_2_N unit cell for monolayer and bilayer C_2_N, respectively. The vacuum region of 15 Å was used to avoid the interactions between two adjacent periodic images. The electronic optimization and structural relaxations were performed until energy and forces converged to 10^−4^ eV (monolayer) and 10^−6^ eV (bilayer) and 0.02 eV/Å, respectively. In order to accurately describe the effect of van der Waals (vdW) interactions, we used the empirical correction method proposed by Grimme (GGA + D2^33^ and GGA + D3^34^) which is a good description of long-range vdW interactions^35–37^. To explore the nature of the long-range Hartree-Fock exchange interactions, the HSE + D3 approaches have been computed. Note that in order to compute bilayer, vdW interactions need to be considered. Finally, the rVV10 nonlocal correlation functional (SCAN + rVV10)^38^ has been computed for comparing our result.

## Results and Discussion

**Figure 1:**
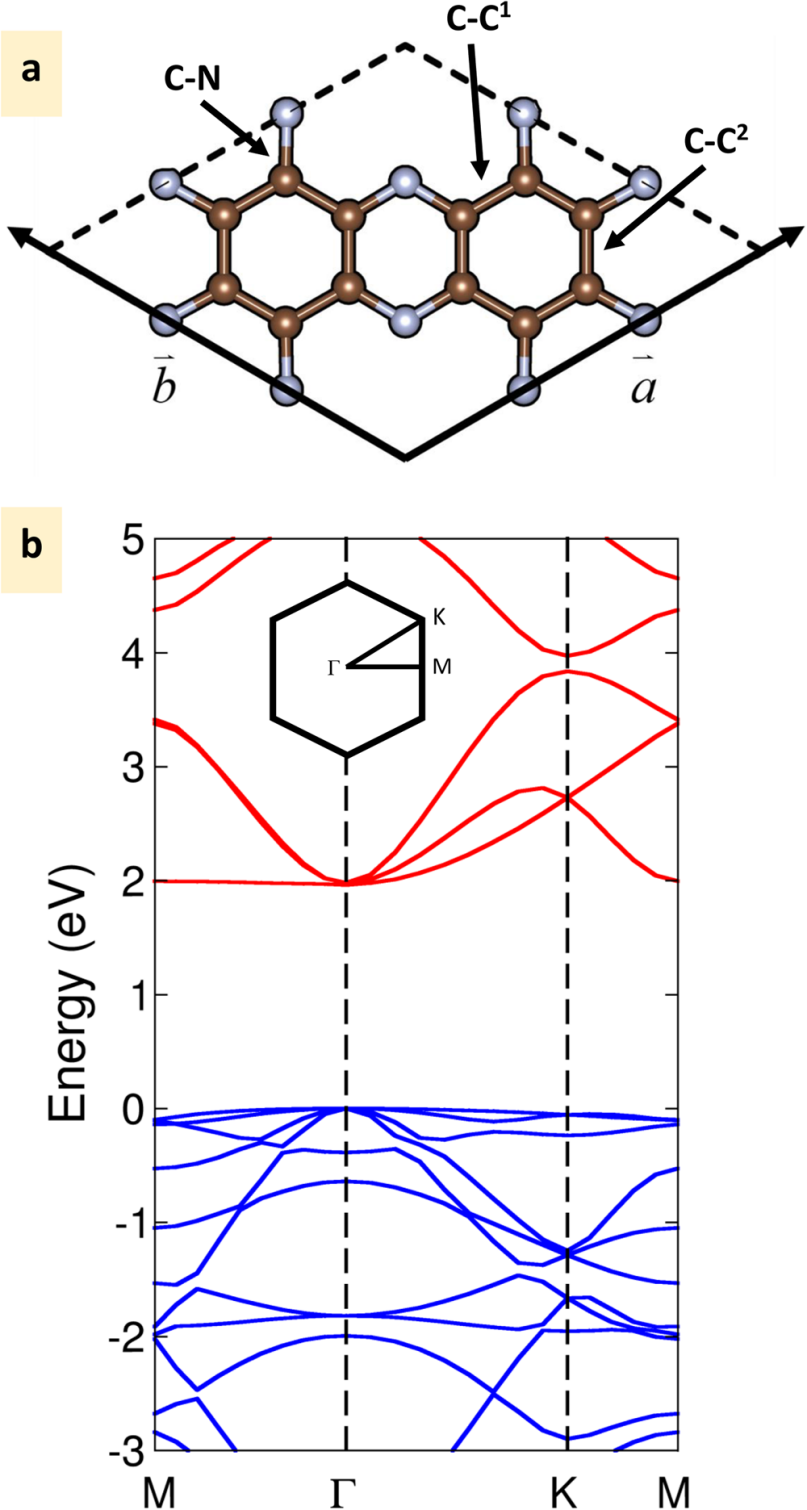
(**a**) Unit cell of monolayer C_2_N crystal structure. C-C^1^ and C-C^2^ are C-C bonds presented in benzene and pyrazine ring, respectively. The C atoms are represented by brown balls and the N atoms are represented by grey balls. (**b**) Calculated band structures of monolayer C_2_N. M(0.5, 0.0, 0.0), Γ(0.0, 0.0, 0.0) and K(1/3, 1/3, 0.0) refer to special points in the first Brillouin zone.

At first, we explored the geometric properties and electronic structure of monolayer C_2_N. A unit cell of C_2_N consists of 12 carbon atoms and 6 nitrogen atoms with uniform holes in the layer. The atomistic ball-stick model of the monolayer C_2_N is illustrated in Fig. 1(a). In C_2_N, there are three types of covalent bonds: the in-plane C-N, C-C^1^ (C-C bonds in benzene rings) and C-C^2^ (C-C bonds in pyrazine rings) as shown in Fig. 1(a). As mentioned before, it is well-known that DFT underestimate band gap attributed to the self-interaction error (SIE), thus we used hybrid exchange-correlation functional to calculate the electronic band structure. Hybrid functionals, which mix a fraction of Hartree-Fock (HF) exchange with local or semilocal exchange, have become increasingly favorite in DFT calculation. In this work, we vary the percentage of HF exchange from the experimental value of *α* = 0.25 to 0.05 in order to mimic the experimental band gap. The mixing parameter 5% can perfectly match the experimental gap. In HSE, the lattice parameter of monolayer C_2_N is 8.317 Å which is little lower than that of GGA values of 8.330 Å^25^. In HSE calculation, the in-plane C-N, C-C^1^ and C-C^2^ bonds (see Fig. 1(a)) are 1.334, 1.468 and 1.427 Å, while the DFT-GGA in-plane C-N, C-C^1^ and C-C^2^ bonds are 1.336, 1.470 and 1.429 Å by Zhang *et al*.^25^ and of 1.337, 1.470 and 1.429 Å by Guan *et al*.^26^, respectively. Note that in all calculations, C-C^1^ bonds are about 3% longer than C-C^2^ bonds. The presence of N atoms results in the small distortion of the benzene rings. The lower of lattice parameter and covalent bond lengths comes from a strong localized wave-function in HSE.

The HSE calculated band structure of monolayer C_2_N is illustrated in Fig. 1(b). Note that in GGA calculation, the distribution of valence band maximum (VBM) and conduction band minimum (CBM) is well separated spatially with a direct energy gap of 1.962 eV; the former mainly contains the nitrogen *p*_*x*_, *p*_*y*_ states and the latter is mainly from the nitrogen *p*_*z*_ state. The *p*_*z*_ states of VBM shift up over *p*_*x*_, *p*_*y*_ when introduced HF exchange as in HSE calculation. Interestingly, when mixing parameter of 5% HF is included, the *p*_*x*_, *p*_*y*_ and *p*_*z*_ band states lie exactly the equal location at the VBM as seen in Fig. S1 in Supplementary information (SI). Since both of the valence and conduction bands are well dispersed and show no localized states to act as recombination centres for the photogenerated electron-hole pairs, C_2_N is good for the separation of photogenerated electron-hole pairs.

**Figure 2:**
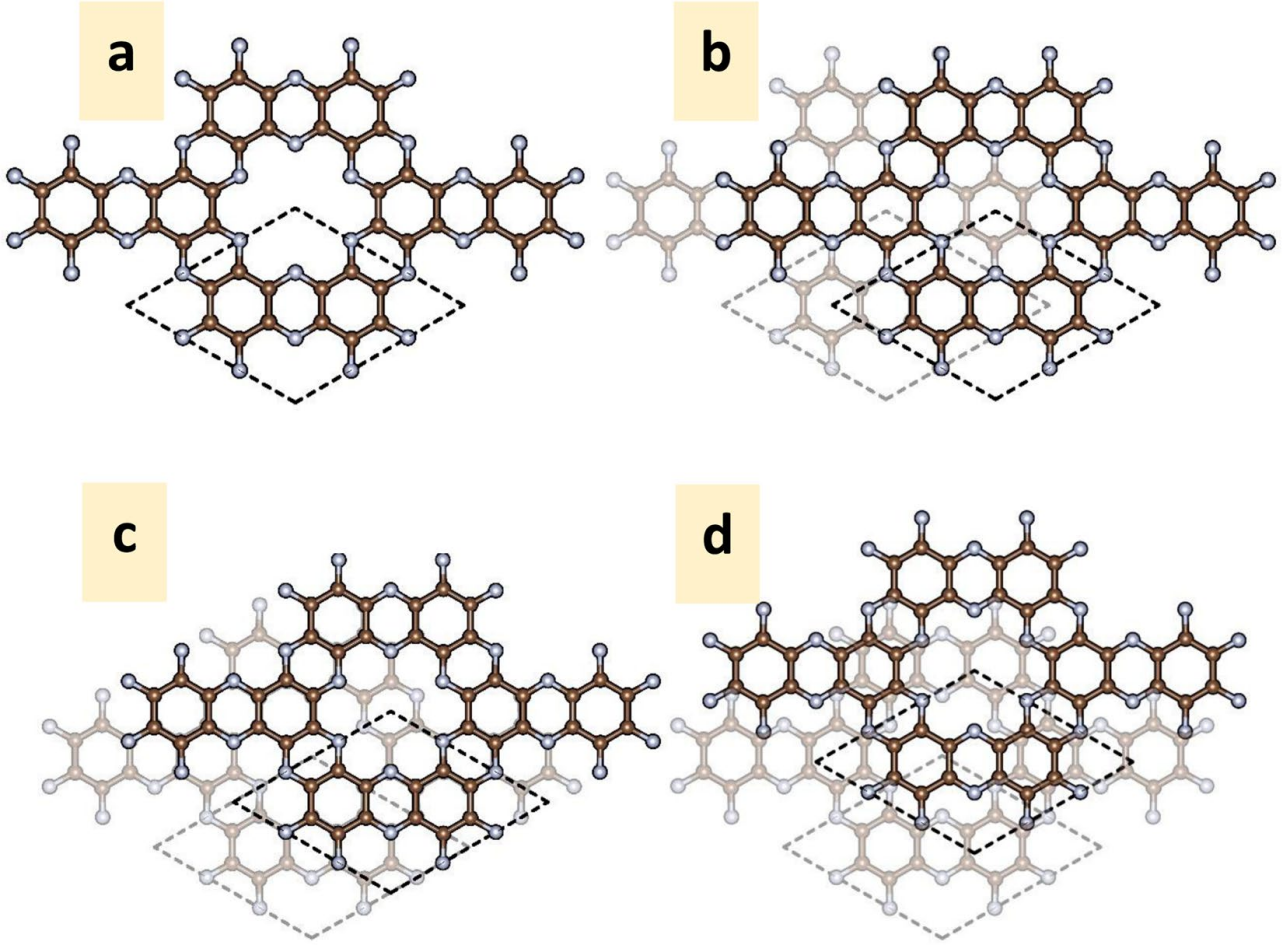
(**a**–**d**) AA-, AB-, AB′ and Min-stacking structures of 2 × 2 supercell bilayer C_2_N, respectively.

Considering the bilayer C_2_N, there are three high-symmetry stacking configurations, which were reported in the previous study by Zhang *et al*.^25^, namely AA, AB and AB′, as shown in Fig. 2(a–c). In AA-stacking, the top layer is stacked directly above the bottom layer. In AB stacking, the top layer is shifted along the zigzag direction by **a**/3-**b**/3, where **a** and **b** are translation vectors of the unit cell. For AB′ stacking, the top layer is shifted along the armchair direction by **a**/2. As presenting in Table 1, the total energy calculations indicate that AB-stacking is the most favorable stacking with the total energy of 13.292 and 2.58 meV/atom lower than that of AA- and AB′-stacking, respectively. Regarding of the previous work by Zhang *et al*.^25^, they proposed three high-symmetry structures including AA, AB and AB′ and suggested that the lowest energy configurations is AB, which agrees with our result. In Zhang *et al*.^25^, the energy difference of AA and AB′ with respect to the energy of AB stacking order are 16 and 3 meV/atom.

**Table 1:** The energy difference relative to the energy of AB stacking order.

Energy difference (meV/atom)			
	GGA + D2		GGA + D3
	Our results	Zhang *et al*.^25^	Our results
AA-AB	13.92	16	15.89
AB′-AB	2.58	3	2.92
Min-AB	−1.81	—	−2.04

The energy of the most stable stacking in Fig. 4 is marked as Min. Note that in previous work by Zhang *et al*.^25^, AB is the minimum configuration.

**Figure 3:**
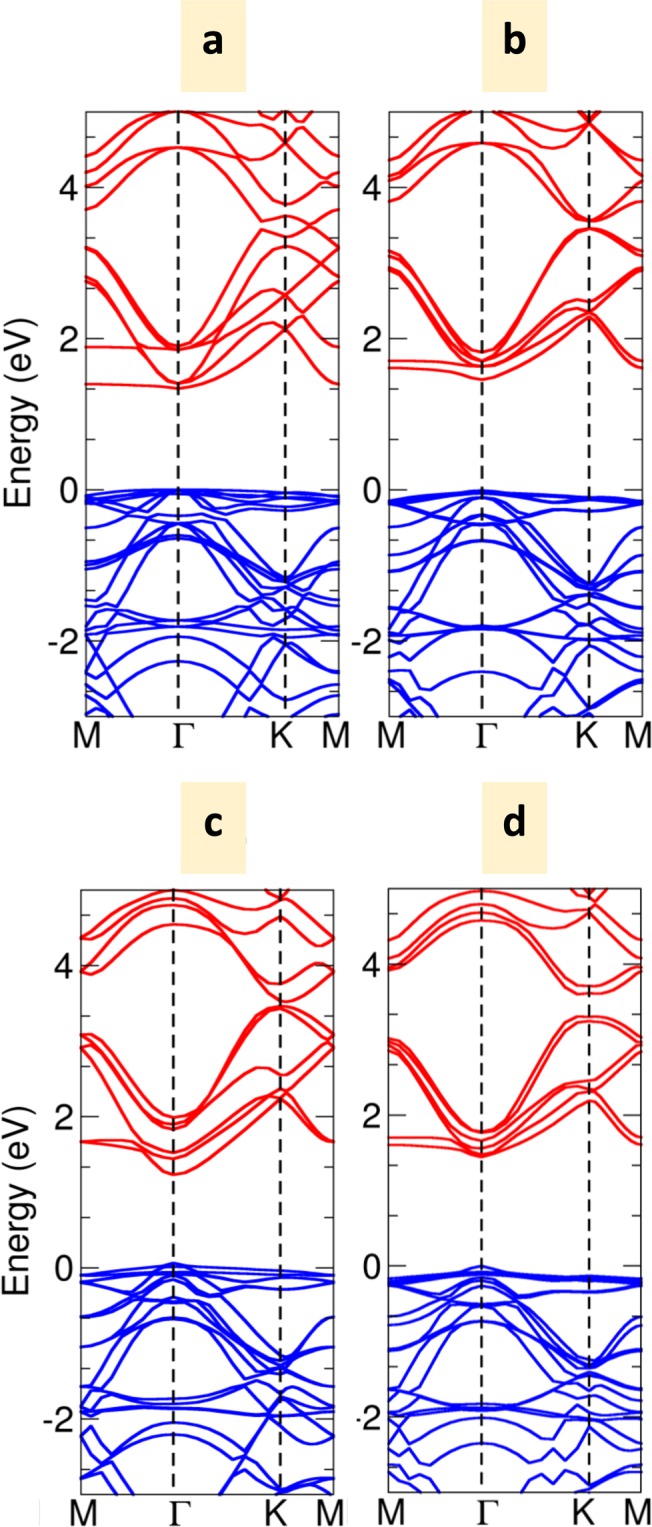
(**a**–**d**) The electronic band structures of AA, AB, AB′ and Min-stacking order, respectively.

The calculated lattice constant (a = |a| = |b|), interlayer distance (*h*_0_), in-plane covalent bond lengths and energy gap of bilayer C_2_N with three different stacking orders are listed in Table 2. In symmetric AA, AB, and AB′ stacking orders, the in-plane covalent bond lengths C-N, C-C^1^ and C-C^2^ show no significant change with different stacking. Note that there is no meaningful difference of in-plane bond lengths between monolayer and bilayer due to the strong covalent bonding. The electronic band structures of bilayer AA, AB, and AB′-stacked C_2_N are shown in Fig. 3(a–c). It is clear that all of them are direct band gap, where VBM and CBM are located at the Γ point. Among these three different stacking orders, the AB-stacked bilayer possesses the widest band gap of 1.467 eV, while for the AA and AB′-stacked bilayer, the band gaps are 1.337 eV and 1.169 eV, respectively. Regarding the previous work by Zhang *et al*.^25^, the band gap of AB-stacking is 1.49 eV, while the band gap of AA and AB′-stacking are 1.34 eV and 1.21 eV. The three high symmetry bilayer configurations retained the direct band gap with different values according to stacking structure. Note that all energy gaps of the three bilayer stacking orders are smaller than the monolayer. The interlayer distance of AA, AB and AB′-stacking are 3.547, 3.203 and 3.242 Å, respectively. Consequently, the most energetically favourable, AB-stacking, gives the lowest interlayer distance.

**Table 2:** The calculated lattice constant, interlayer distance, the in-plane covalent bond lengths and energy gap of bilayer C_2_N with different stacking orders.

	a(Å)	*h*_0_	C-N	C-C^1^	C-C^2^	*E*_*g*_(eV)
AA	8.333	3.547	1.336	1.471	1.430	1.337
AB	8.333	3.203	1.336	1.470	1.430	1.467
AB′	8.333	3.242	1.337	1.469	1.430	1.169
Min	8.333	3.099	1.336	1.469	1.430	1.444

**Figure 4:**
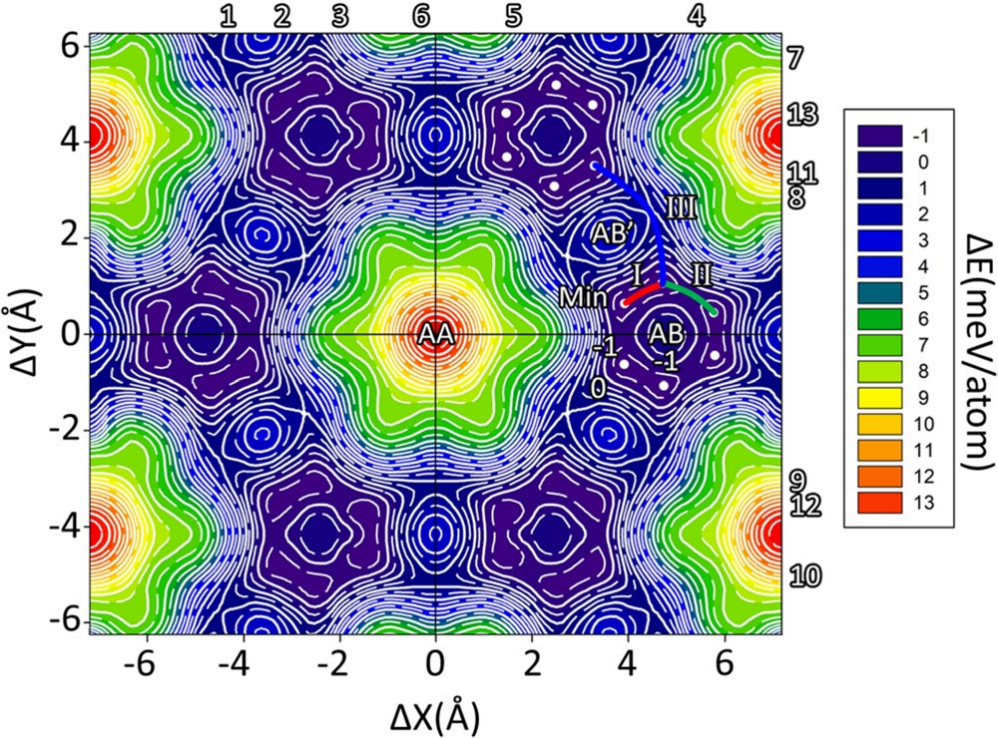
2D-energy profile surface of bilayer C_2_N depending on the inter-layer relative slide distance (Δ*X*, Δ*Y*). The colour scale and contours represent the energy per atom relative to the energy per atom of AB. The solid contour line is 1 meV/atom increment. The minimum energy points are represented by white dots. Path I, Path II, Path III are the translation paths between two minimum point of intra-region, inter-region and cross over region, respectively.

In this work, a more comprehensive energy landscape has been investigated in order to explore other possible equilibrium structures of bilayer C_2_N apart from the three proposed structures. We strive for more energetically stable configurations by performing two-dimensional energy scanning along both zigzag (Δ*X*) and armchair (Δ*Y*) directions. The AA, AB, and AB′ stacking orders are equivalent to the shift of (Δ*X* = 0, Δ*Y* = 0), (Δ*X* = 4.811 Å, Δ*Y* = 0), and (Δ*X* = 3.609 Å, Δ*Y* = 2.084 Å), respectively. For every given shift, the atomic position and the interlayer distance of the bilayer are allowed to relax. Figure 4 shows the 2D energy surface of bilayer C_2_N, referenced to AB stacking, as a function of relative shift (Δ*X*, Δ*Y*). The discrete set of the sliding energy were interpolated to finer mesh by using Renka II procedure^39–41^. In Fig. 4, it is clearly shown that the high symmetry AB-stacking is not the most favourable stable configuration. Interestingly, there are six energetically favourable configurations surrounding the AB stacking. More specifically there are three symmetric regions where the energy is lower than the AB configuration. In each region, there are two actual minimum points (Min) marked as white dot in Fig. 4. The energy difference between Min and AB point in our calculation is approximately 1.81 meV/atom. The different choice of dispersion correction (GGA + D3) is also considered. Surprisingly, the actual minimum points calculated from GGA + D3 are all exactly the same position as GGA + D2 which shown in Fig. S2 in SI.

The energy differences of the symmetric AA, AB, AB′ and Min point stacking with respect to the energy of AB-stacking are shown in Table 1. The calculated energy difference confirmed that the AB stacking configuration is not the most favourable structure for bilayer C_2_N. The local structure of one of the minimum energy points (Min) is shown in Fig. 2(d). Regarding the symmetric AA, AB, and AB′-stacking, all atoms on the top layer are located directly above the bottom layer. In Min point stacking of Fig. 2(d), all atoms on the top layer are not located directly above those of the bottom layer. The Min configuration is slightly shifted from the high symmetry AB-stacking structure. Unlike the symmetric AA, AB, and AB′-stacking, the in-plane covalent bond lengths C-N, C-C^1^ and C-C^2^ of the Min point are not equal, so we refer to the average bond length. The average bond length of C-C^1^, C-C^2^ and C-N are 1.469, 1.430 and 1.336 Å, respectively. The interlayer distance (*h*_0_) is 3.099 Å which is slightly lower than that of the AB-stacking structure. The average bond length and interlayer distance are listed in Table 2.

The electronic band structure corresponding to the configuration in Fig. 2(d) is presented in Fig. 3(d). Since the Min configurations are surrounding the AB stacking configuration, the calculated band structure of Min point looks similar to high-symmetry AB stacking order. The forbidden gap is still the direct-gap with a band gap of 1.444 eV. However, the band gap is lower than that of the high-symmetry AB stacking order by 23 meV.

**Figure 5:**
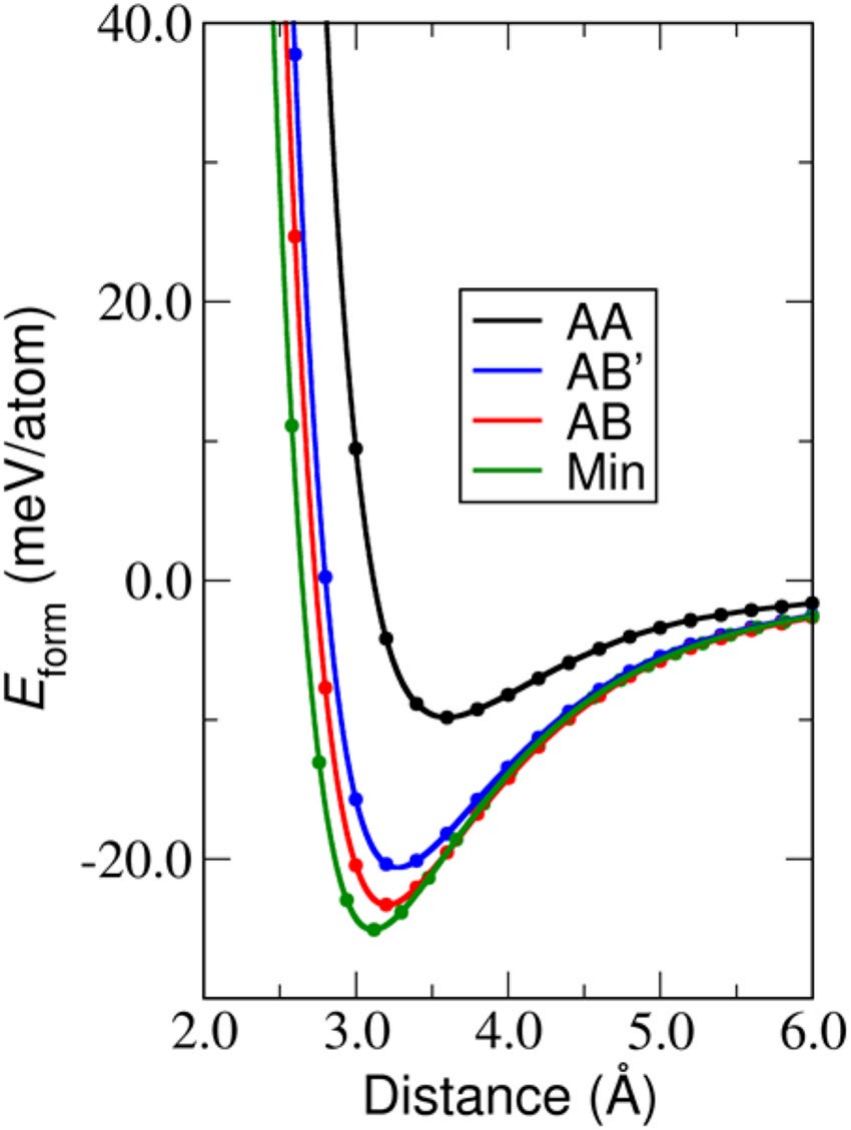
The formation energy (*E*_*form*_) of each stacking order as a function of inter-layer distance.

The stability of the bilayer can be represented in terms of the formation of energy. We calculated the formation energy per atom (*E*_*form*_) as a function of adjacent layer distance for each stacking order based on the GGA + D2 functional as shown in Fig. 5. The formation energy is defined as the total energy difference per atom between the bilayer and the component of two monolayers which are separated with adequate distance (*E*_*form*_ = *E*_*bilayer*_ − 2*E*_*monolayer*_). The formation energy is the energy required to produce a bilayer from monolayers. The structures of bilayer C_2_N have similar trends of formation energy per atom. In Fig. 5, the Min stacking possesses the lowest formation energy of −23.85 meV/atom with the minimum interlayer distance of 3.099 Å. For the three high-symmetry stacking, AA, AB′ and AB-stacking, the formation energies are −9.8 −20 and −23 meV/atom which correspond the minimum interlayer distance of 3.547, 3.242 and 3.203 Å respectively. This demonstrates that the Min stacking configuration is the most favourable in term of stability. In order to speed up the exploration of exchange-correlation calculation (HSE + D3, SCAN + rVV10), we adopted the following Min-stacking configuration from GGA + D2/GGA + D3 to calculate the formation energy. The formation energy of AA, AB′, AB and Min-stacking of HSE + D3 and SCAN + rVV10 are shown in Fig. S3 in SI. It is clear that the formation energy of the Min position are lower than that of AB position in both HSE + D3 and SCAN + rVV10. These mean there are truly more favorable structure than AB.

**Figure 6:**
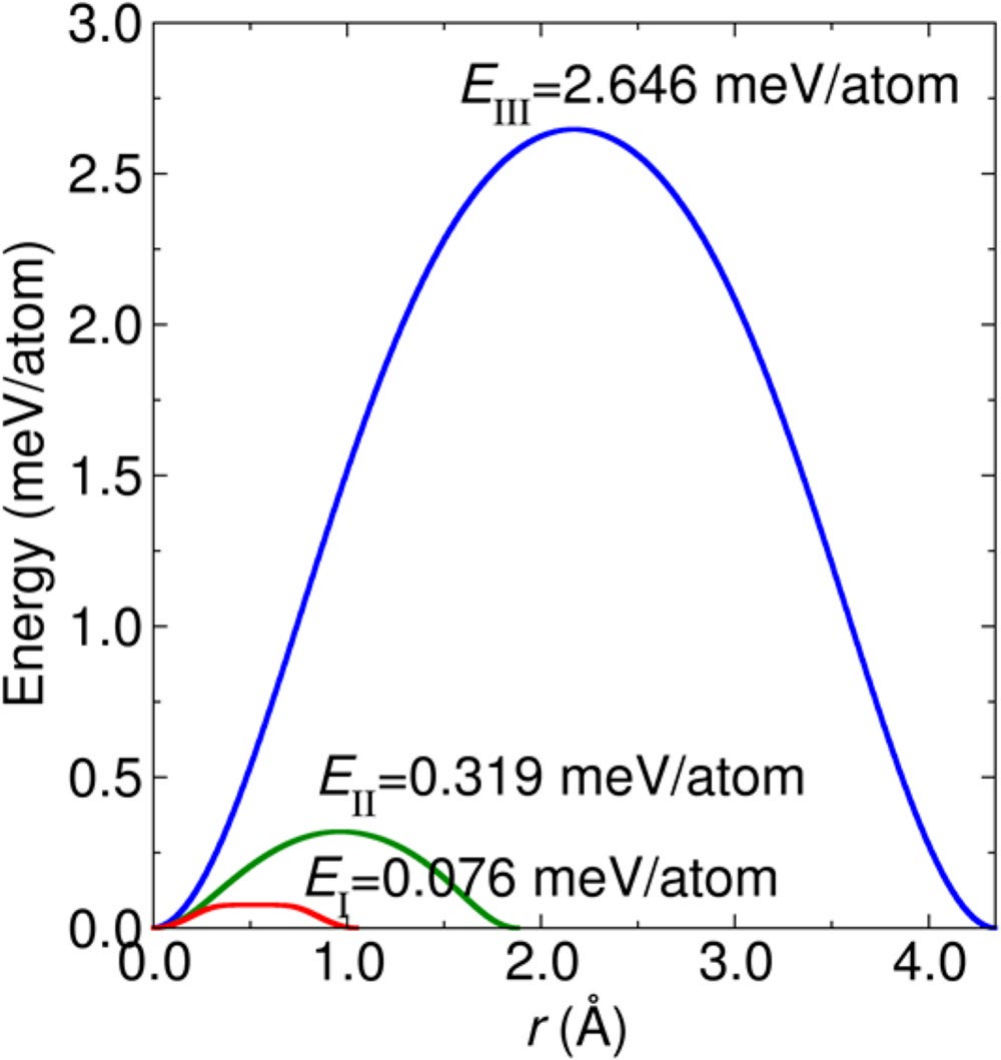
Translated energy barrier as a function of distance *r* between two minimum points along the Path I, II and III. The unit of energy barrier and distance *r* are meV/atom and Å, respectively.

In Fig. 4 with deliberating at the Min points, the sliding paths between the Min points can be considered. There are three possible sliding paths. The first one is connected between two local minimum points in the same region called intra-region Path I. The second path is connected between two local minimum points in different regions called inter-region Path II. The third path is connected between two local minimum points between cross-over regions called Path III. The translated energy barrier demonstrates how difficult it is from one stacking to another by sliding one layer along a specific path. The translated energy barrier corresponding to path I, II and III as a function of distance *r* of the two local minimum points are shown in Fig. 6. Obviously, it is difficult to slide along path III. However, it might be possible that there is a transformation from two minimum point in path I and path II because they require small amount of energies of 0.076 and 0.319 meV/atom to overcome these energy barriers.

Next, imaging the existing forces holding two layers together act like springs, linking one C_2_N layer to the next layer, it is possible to calculate the spring constant (*k*) at the minimum structure of bilayer C_2_N. For estimation, the spring constant in zigzag and armchair direction are equal at the Min point. Along armchair direction, the energy-strain relation equation can be fitted with the equation *E*(*x*) = *kx*^2^/2, where *x* is the displacement from Min Point. In this case, the fitted spring constant value is 0.0859 N/m per atom. This solution leads us to find the force and the angular frequency of the most stable C_2_N bilayer structure. In this present work, the calculated frequency of the interlayer shear mode around local minimum point is approximately 2.02 × 10^12^ rad/s or roughly 0.322 THz.

## Summary

In summary, we performed first-principles calculations to explore the stacking stability and electronic properties of C_2_N bilayer. The stacking order and electronic band structure of three high-symmetry stacking configurations namely as AA, AB and AB′-stacking were calculated. Among these three high-symmetry stacking configurations, AB-stacking is the most favourable with smallest interlayer distance but highest band gap. Our results agree well with the previous study^25^. More comprehensive energy landscape has been studied to explore other possible configurations. We however found that there are three regions around AB which provide truly lower energy per atom than AB-stacking structures. In these regions, all atoms on the top layer are shifted from the bottom layer. Fascinatingly, the electronic band structures of the stable configuration express the same fashion as the high-symmetry AB stacking but they differ from AB-stacking in term of the energy gap and band line splitting. Importantly, the energy gap can be modified according to the stacking order. In terms of stability, the formation energy (*E*_*form*_) per atom confirms the fact that these regions are more stable than AB stacking. Lastly, the frequency of the interlayer shear mode was determined to be 2.02 × 10^12^ rad/s or 0.322 THz.

## Supplementary information

Supplementary information
