# Author Correction: Stacking stability of C_2_N bilayer nanosheet

**DOI:** 10.1038/s41598-020-60959-7

**Published:** 2020-03-03

**Authors:** Klichchupong Dabsamut, Jiraroj T-Thienprasert, Sirichok Jungthawan, Adisak Boonchun

**Affiliations:** 1 Department of Physics, Faculty of Science, Kasetsart University, Bangkok, 10900 Thailand; 2 Thailand Center of Excellence in Physics, Commission on the Higher Education, 328 Si Ayutthaya Road, Bangkok, 10400 Thailand; 3 School of Physics, Institute of Science, and Center of Excellence in Advanced Functional Materials, Suranaree University of Technology, Nakhon Ratchasima, 30000 Thailand

Correction to: *Scientific Reports* 10.1038/s41598-019-43363-8, published online 02 May 2019

This Article contains errors.

The paper does not discuss the related work of R. Longuinhos and J. Ribeiro-Soares^1^; as a result the Introduction should also include the following:

“Recently for bilayer C2N, R. Longuinhos and J. Ribeiro-Soares^1^ proposed several lower-symmetry stacking configurations which are AB’2, AB’3, AB’4 and AB”. They suggested that among these lower-symmetry stackings, AB” is the most stable position in equilibrium under standard conditions… According to energy landscape, our most stable stacking configuration named Min-stacking is accidentally the same structure as AB” in the previous study by R. Longuinhos and J. Ribeiro-Soares^1^”

In addition, the Results and Discussion reads:

“The energy differences of the symmetric AA, AB, AB’ and Min point stacking with respect to the energy of AB-stacking are shown in Table1. The calculated energy difference confirmed that the AB stacking configuration is not the most favourable structure for bilayer C2N. The local structure of one of the minimum energy points (Min) is shown in Figure2(d)… The forbidden gap is still the direct-gap with a band gap of 1.444 eV.”

and should instead read:

“The energy differences of the symmetric AA, AB, AB’ and Min point stacking with respect to the energy of AB-stacking are shown in Table 1. The calculated energy difference confirmed that the AB stacking configuration is not the most favourable structure for bilayer C2N. The local structure of one of the minimum energy points (Min) is shown in Figure2(d), which is the same structure as AB” stacking of the previous study^1^… The forbidden gap is still the direct-gap with a band gap of 1.444 eV, which is higher than that of 1.3 eV in previous work with DFT-LDA.^1^”
