# Supplementary material for: Stacking stability of C2N bilayer nanosheet
Source: Sci Rep. 2019 May 2;9:6861. doi: 10.1038/s41598-019-43363-8 (PMC6497902; doi:10.1038/s41598-019-43363-8)
Supplement: Supplementary file 1 — Supplementary information [file 41598_2019_43363_MOESM1_ESM.docx]

**Supplementary information for**

**“Stacking stability of C_2_N bilayer nanosheet”**

Klichchupong Dabsamut,^1,2^ Jiraroj T-Thienprasert^1,2^ Sirichok Jungthawan,^2,3^ and Adisak Boonchun*^1,2^

^1^Department of physics, Faculty of science, Kasetsart University, Bangkok 10900, Thailand.

^2^ Thailand Center of Excellence in Physics, Commission on the Higher Education, 328

Si Ayutthaya Road, Bangkok 10400, Thailand

^3^School of Physics, Institute of Science, and Center of Excellence in Advanced Functional Materials, Suranaree University of Technology, Nakhon Ratchasima, 30000, Thailand

*E-mail: adisak.bo@ku.th, fsciasb@ku.ac.th


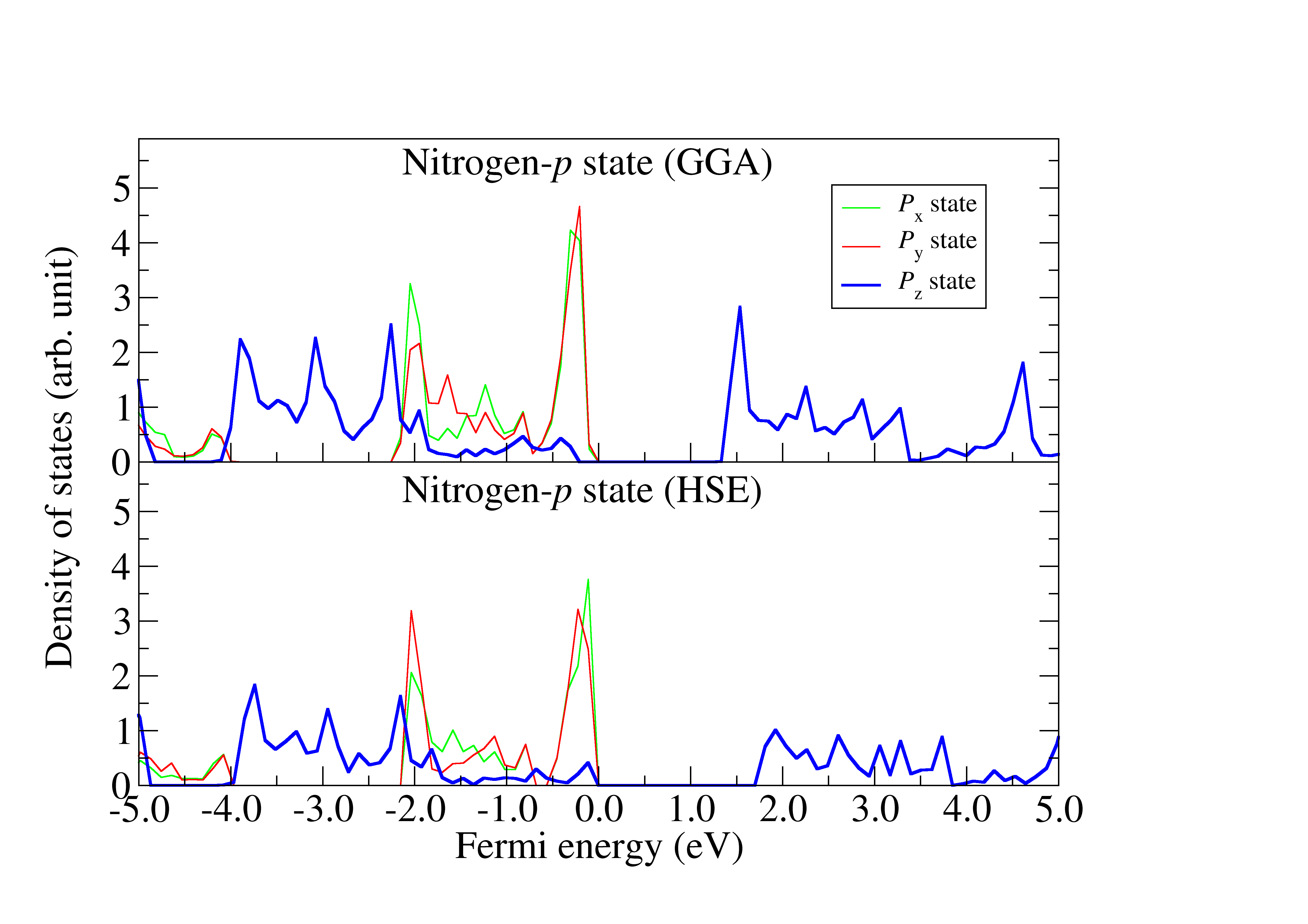


**Figure S1** Calculated projected density of states of nitrogen *p*-orbitals of monolayer C_2_N. The energy of valence band maximum (VBM) is set to zero.

S1


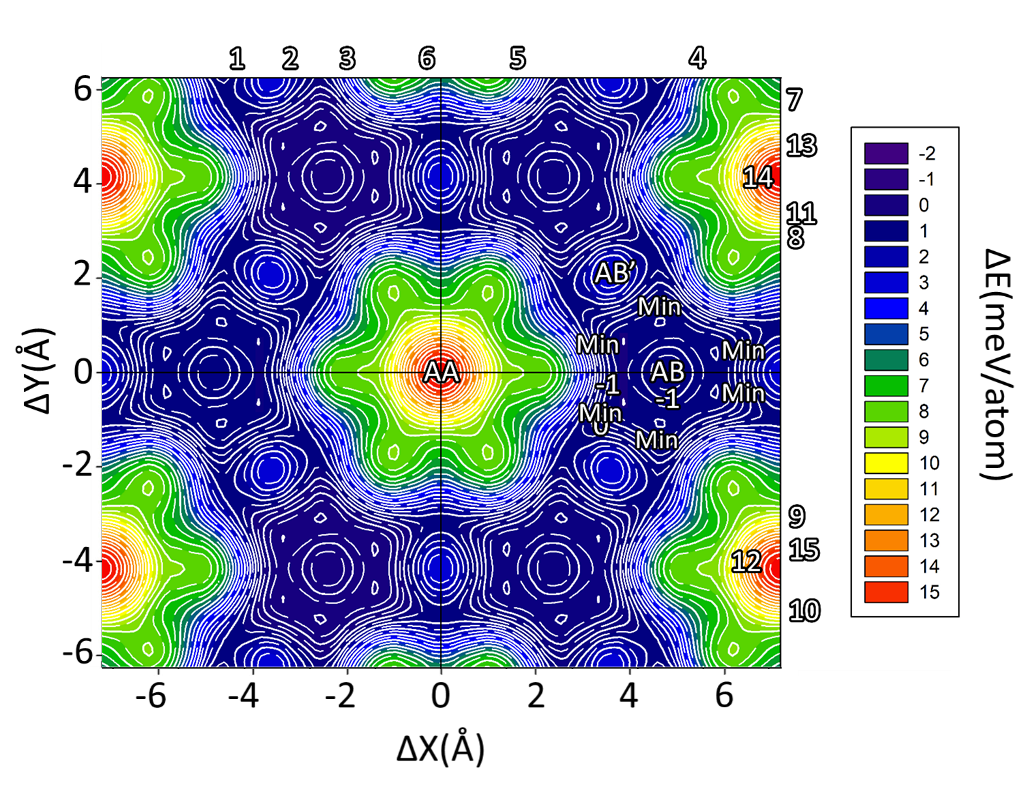


S2

**Figure S2** 2D-energy profile surface of bilayer C_2_N depending on the inter-layer relative slide distance (∆X, ∆Y) using **GGA+D3** calculation. The color scale and contours represent the energy per atom relative to the energy per atom of AB. The solid contour line is 1 meV/atom increment. Similar to GGA+D2 calculation, the minimum energy point is slightly shifted from high symmetry AB-stacking structure.


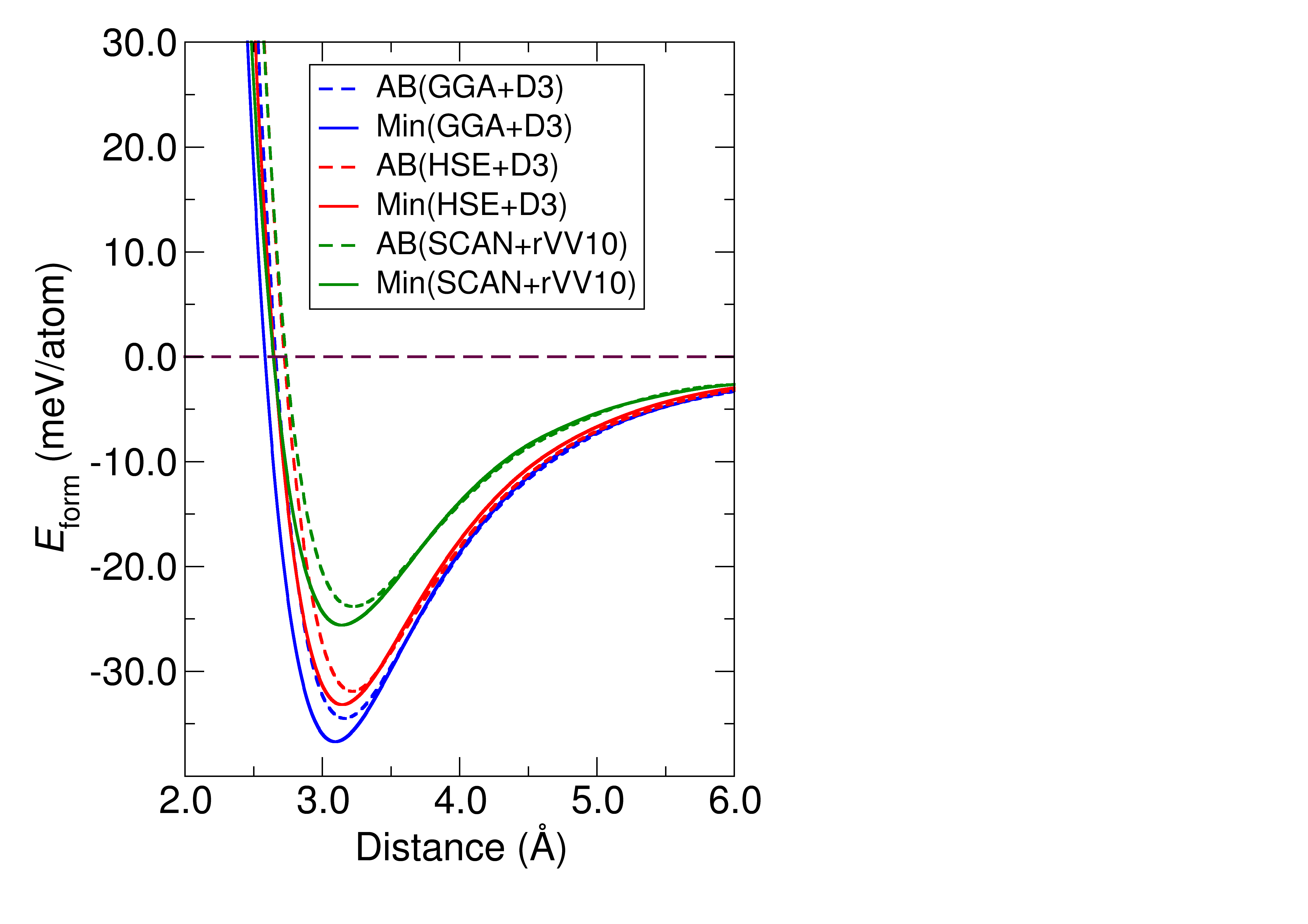


**Figure S3** The formation energy (*E*_form_) of AB- and Min-stacking configuration in other dispersion correction (GGA+D3), the long-range Hatree-Fock exchange interaction (HSE+D3) and the rVV10 with nonlocal correlation functional (SCAN+rVV10).

S3
